# Evaluation and verification of a simplified lead equivalency measurement method

**DOI:** 10.1002/acm2.12810

**Published:** 2020-01-08

**Authors:** Richard Ryan Wargo, Areej Fawzi Aljabal, Pei‐Jan Paul Lin

**Affiliations:** ^1^ Department of Radiology Virginia Commonwealth University Richmond VA USA; ^2^ Division of Medical Physics Department of Radiation Oncology Virginia Commonwealth University Richmond VA USA

**Keywords:** attenuation measurement, lead and nonlead protection apparel, lead equivalence, lead foil tape

## Abstract

**Purpose:**

This technical note presents an inexpensive tool and method for determining lead equivalency using digital radiography x‐ray equipment.

**Methods:**

A test tool was developed using commercially available lead tape (3M™ Lead Foil Tape 421). The test tool consisted of nine varying lead thick squares arranged in a larger square (0.1, 0.2, 0.3, 0.4, 0.5, 0.6, 0.7, 0.8, and 1.0 mm). It was imaged on a DR plate with a digital portable x‐ray unit across a range of energies (60–120 kVp) and two beam filtrations. Lead equivalency was determined by using the linear relationship between dose to the detector and pixel values in the raw images. The lead equivalency of the tape was validated using known lead thicknesses (physically measured with caliper). Additional lead equivalency measurements were made for protective eyewear, a thyroid shield, and a lead apron.

**Results:**

The test tool and method measured the two known lead thicknesses to be –9.7% to 7.1% different from the actual values across the range of energies under normal x‐ray beam conditions and under a 1‐mm copper filtered x‐ray beam. The additional lead equivalency measurements of radiation protection apparel across energies ranged from –6% to 20% for both beam conditions when compared with the values provided by the manufacturer.

**Conclusion:**

This work validates the test tool and methodology as an inexpensive alternative to checking the lead equivalency of radiation protection apparel in a clinical setting. The methodology is equipment independent with a few prerequisites.

## 1 INTRODUCTION

The importance of radiation protection has been well documented under the current assumption of the linear no‐threshold (LNT) model.^1^, ^2^, ^3^ Despite the LNT model limitations, its purpose is to safely address the risk associated with exposure to radiation. Three main principles that are always emphasized with regards to radiation protection are time, distance, and shielding. Radiation protection apparel is used widely in the medical environment to provide shielding against primary and scattered radiation for patients and radiation workers. One of the main concerns with radiation protection apparel is their attenuation properties.^4^, ^5^, ^6^, ^7^, ^8^ With the use of nonlead radiation shielding materials, it is often of importance to know the attenuation properties of the radiation protection apparel in question. Independently verifying vendors' lead equivalence claims can be a daunting task. There are a few standards available that address how to measure the attenuation properties of this radiation protection apparel:

- ASTM F 2547‐18 Standard test method for determining the attenuation properties in a primary x‐ray beam of materials used to protect against radiation generated during the use of x‐ray equipment^9^
- ASTM F 3094‐14 Standard test method for determining protection provided by x‐ray shielding garments used in medical x‐ray fluoroscopy from sources of scattered x‐rays^10^
- IEC 61331‐1:2014 Protective devices against diagnostic medical x‐radiation — Part 1: Determination of attenuation properties of materials 11

However, the ability for people, companies, and institutions to independently verify the lead equivalency of shielding materials using these standards can be cumbersome and requires specialized equipment for setup and measurement. This study investigates the development of an inexpensive test tool using lead tape and a process to determine the lead equivalency of materials using digital x‐ray equipment.

## 2 MATERIALS AND METHODS

**Figure 1:**
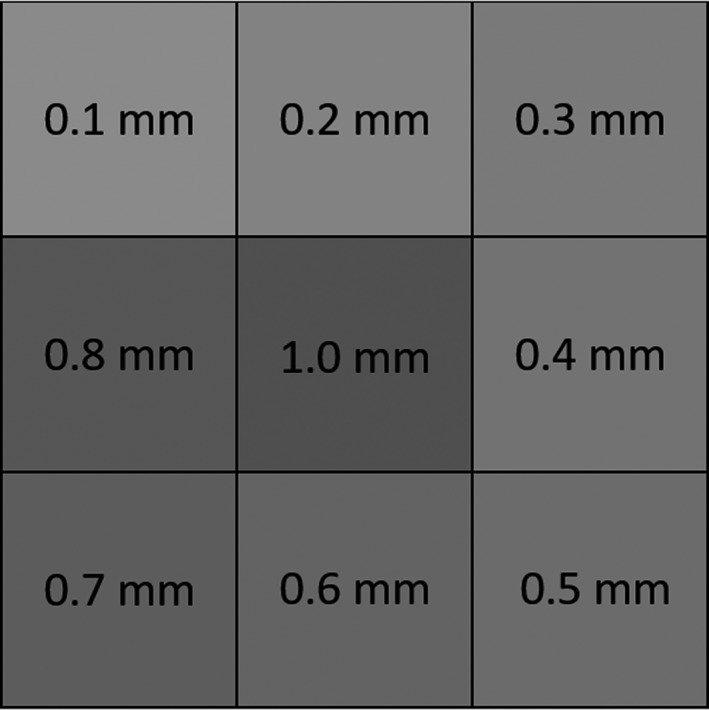
Drawing of test tool using lead 3M Lead Foil Tape 421.

To measure the lead equivalency of objects, there are some basic requirements. In the simplest iteration, one needs an x‐ray source, test object, and a radiation detector. The first part of our setup was developing a test tool using commercially available lead tape — 3M^TM^ Lead Foil Tape 421 (3M Corporate, St. Paul, MN). The lead foil tape’s total thickness is 6.3 mil (0.16 mm) which is comprised of 4.0 mil (0.1 mm) lead foil backing and 2.3 mil (.06 mm) of rubber adhesive. The lead foil was cut into 2.5 cm squares to create a 3 by 3 (7.5 cm by 7.5 cm) square of increasing lead thicknesses (0.1, 0.2, 0.3, 0.4, 0.5, 0.6, 0.7, 0.8, and 1.0 mm) [Fig. 1]. These were attached to a clear film to act as the supporting base.

A portable digital x‐ray system (Carestream DRX Revolution; Carestream DRX Plus 3543C) was used to make the measurements. Before starting to use the test tool to evaluate radiation protection garments, there were some initial tests done to reduce potential errors and problems in the process. The tube voltage accuracy and exposure reproducibility were evaluated using a RTI Black Piranha Model 657 (RTI, Towaco, NJ, USA). The tube voltage accuracy was within 1.5% across the energy range used. The exposure reproducibility had a coefficient of variation of under 0.2%. Next, the linearity and uniformity of the digital detector were evaluated using a range of exposures (between 0 uGy and 50 uGy) at 70 kVp with 1 mm Cu in the beam and a source‐to‐detector distance of 150 cm.

The setup for validating the lead foil tape and measuring the lead equivalence of the different radiation protection apparel was at a source‐to‐image distance of 150 cm with the detector on the floor. To validate the lead foil tape, known lead thicknesses were used. Pieces of 1/32” and 1/64” lead were laid on the digital detector with the test tool placed in the center. There are typically tolerances of +/− 0.005” (0.127 mm) with regards to commercially available lead.^12^, ^13^ The thickness of the lead was determined using a caliper. Similarly, the different radiation protection apparel (lead glasses (lead lenses), side shield of lead glasses (lead with vinyl sheet), thyroid (composite — antimony and lead), and apron (composite — antimony and lead)) were laid flat on the digital detector and exposed all at once. Exposures were made in increments of 10 from 60 kV to 120 kV under two beam conditions (no filter and 1 mm Cu filter). Table 1 shows the beam quality measured by the RTI Black Piranha for the portable digital x‐ray system used.

**Table 1:** Carestream DRX revolution beam quality.

X‐ray tube voltage (kV)	HVL (mm Al)
60	2.50
70	2.96
80	3.42
90	3.85
100	4.35
110	4.89
120	5.37

**Figure 2:**
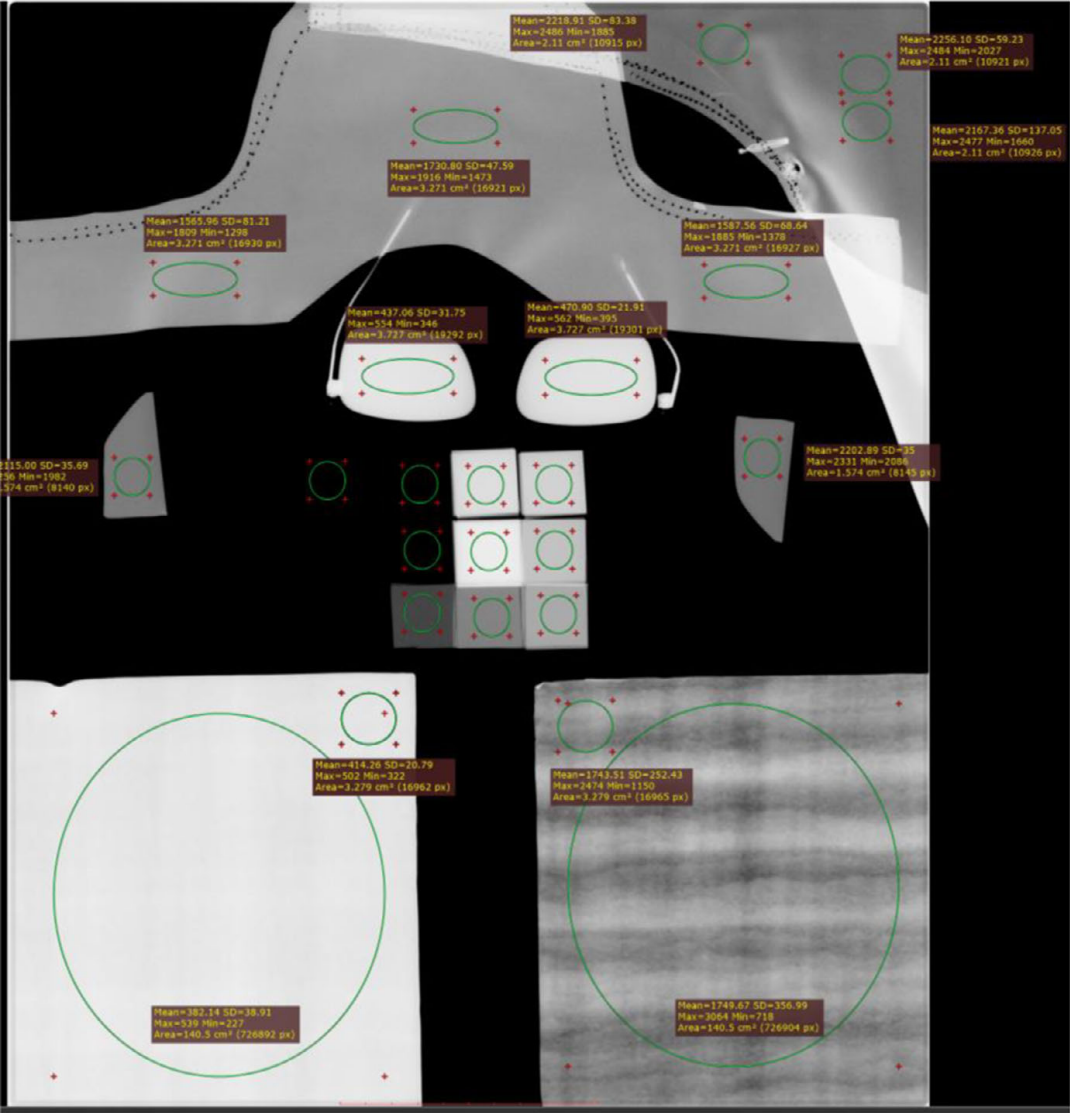
Digital radiographic image of test tool, radiation protection apparel, and lead sheets.

The “FOR PROCESSING” images were exported off the system and evaluated on a computer with RadiAnt DICOM Viewer (Poznań, Poland). For validation of the lead foil tape, region of interest (ROI) measurements resulting in the mean pixel values (MPV) were made on the lead sheets and lead tape. ROI measurements were also placed on the radiation protection apparel [Fig. 2]. The lead equivalences were calculated by fitting the transmission data to Archer’s equation^14^, ^15^ using the curve fitting tool from MATLAB^®^ (MathWorks, MA, USA) and by linear interpolation using the MPV:

(1) $$B={\left[\left(1+\frac{\beta }{\alpha }\right)e^{\alpha \gamma x}-\frac{B}{\alpha }\right]}^{-\frac{1}{\gamma }}$$

(2) $$x=\frac{1}{\alpha \gamma }\ln\left(\frac{B^{-\gamma }+\frac{\beta }{\alpha }}{1+\frac{\beta }{\alpha }}\right)$$

Here *B* represents the transmission of x‐rays for a given thickness, *x*, of shielding materials in mm. *α, β*, and *γ* are fitting parameters for Archer’s eq. (1). Knowing B, α, β, and γ, eq. (2) can be used to calculate the lead thicknesses, which can then be compared with the known lead thicknesses. The calculated lead equivalencies of the radiation protection apparel were compared with the values supplied by the vendors and manufacturers.

## 3 RESULTS

Table 2 shows the results of the caliper measurements of the lead sheets used for validation of the test tool. Table 3 shows the results of the detector uniformity at different detector air kerma levels. Table 4 has the fitting parameters results from fitting the transmission data to Archer’s equation. Table 5 shows the calculated lead thicknesses against the caliper averaged lead thicknesses of the lead sheets. Table 6 shows the calculated lead equivalencies for the different radiation protection apparel and the percent error with regards to the vendor's/manufacturer's claimed lead equivalency.

**Table 2:** Caliper measurements for lead sheets.

Commercial lead sheets	Side 1 (mm)	Side 2 (mm)	Side 3 (mm)	Side 4 (mm)	Average (mm)
Pb – 1/32”(0.794 mm)	0.880	0.885	0.890	0.905	0.894
	0.900	0.890	0.905	0.895	
Pb – 1/64”(0.397 mm)	0.410	0.460	0.470	0.395	0.424
	0.420	0.420	0.380	0.440	

Lead sheets used for validation of the test tool were measured using a caliper.

**Table 3:** Detector uniformity.

ROI position	A (UL)	B (UR)	C (C)	D (LL)	E (LR)	CV	Detector air kerma (μGy)
Mean pixel value	1450	1404	1455	1442	1409	1.7%	1.1
	13031	12617	13118	13035	12725	1.7%	10.3
	14328	13876	14429	14340	13997	1.7%	11.4
	29013	28099	29210	29037	28354	1.7%	23.2

C, Center; CV, Coefficient of Variation; LL, Lower Left; LR, Lower Right; UL, Upper Left; UR, Upper Right.

Uniformity was evaluated objectively and visually at varying detector air kerma levels.

**Table 4:** Archer’s equation fitting parameters for transmission curves corresponding to varying tube voltages.

kV	60	70	80	90	100	110	120
α	4.528	4.678	3.723	3.170	3.032	2.983	2.937
β	14.553	11.717	9.919	8.662	7.509	6.597	5.930
γ	0.474	0.755	0.875	0.937	0.969	0.987	0.996

**Table 5:** Calculated versus measured lead thickness for lead sheets using test tool

Output	kV	DAK (μGy)	kV	DAK (μGy)	kV	DAK (μGy)	kV	DAK (μGy)	kV	DAK (μGy)	kV	DAK (μGy)	kV	DAK (μGy)
	60	21.6	70	19.7	80	19.3	90	21.5	100	18.5	110	19.5	120	22.8
Calculation method	AF (mm)	LI (mm)	AF (mm)	LI (mm)	AF (mm)	LI (mm)	AF (mm)	LI (mm)	AF (mm)	LI (mm)	AF (mm)	LI (mm)	AF (mm)	LI (mm)
Pb ‐ 1/32" (0.894 mm)	0.807	0.957	0.875	0.930	0.874	0.915	0.878	0.913	0.886	0.920	0.892	0.927	0.898	0.933
Pb ‐ 1/64" (0.424 mm)	0.445	0.445	0.439	0.443	0.438	0.440	0.438	0.440	0.437	0.438	0.436	0.437	0.436	0.437
Percent Error	−9.7%	7.1%	−2.1%	4.1%	−2.2%	2.4%	−1.8%	2.2%	−0.9%	2.9%	−0.2%	3.7%	0.5%	4.4%
	4.9%	4.9%	3.4%	4.4%	3.2%	3.7%	3.2%	3.7%	3.0%	3.2%	2.7%	3.0%	2.7%	3.0%

AF, Archer’s Fit; DAK, Detector Air Kerma; LI, Linear Interpolation.

**Table 6:** Calculated lead equivalencies for radiation protection apparel using test tool.

Output	kV	DAK (μGy)	kV	DAK (μGy)	kV	DAK (μGy)	kV	DAK (μGy)	kV	DAK (μGy)	kV	DAK (μGy)	kV	DAK (μGy)
	60	21.6	70	19.7	80	19.3	90	21.5	100	18.5	110	19.5	120	22.8
Calculation method	AF (mm)	LI (mm)	AF (mm)	LI (mm)	AF (mm)	LI (mm)	AF (mm)	LI (mm)	AF (mm)	LI (mm)	AF (mm)	LI (mm)	AF (mm)	LI (mm)
Glasses — Lenses (L) [0.75 mm PbEq]	0.65	0.72	0.77	0.80	0.82	0.85	0.83	0.86	0.84	0.87	0.85	0.88	0.86	0.89
	−*13.1%*	−*4.3%*	*2.3%*	*7.2%*	*8.7%*	*13.1%*	*11.2%*	*15.2%*	*12.3%*	*16.4%*	*13.2%*	*17.5%*	*14.0%*	*18.3%*
Glasses — Lenses (R) [0.75 mm PbEq]	0.68	0.78	0.77	0.81	0.81	0.84	0.82	0.85	0.82	0.85	0.83	0.85	0.83	0.86
	−*9.7%*	*3.7%*	*2.9%*	*8.1%*	*7.3%*	*11.5%*	*9.2%*	*12.7%*	*9.7%*	*13.2%*	*10.1%*	*13.6%*	*10.4%*	*14.0%*
Glasses — Side Shield (L) [0.35 mm PbEq]	0.37	0.38	0.37	0.38	0.38	0.38	0.38	0.38	0.38	0.38	0.39	0.39	0.39	0.39
	*6.3%*	*7.1%*	*6.6%*	*7.7%*	*7.7%*	*8.6%*	*8.6%*	*9.1%*	*9.4%*	*9.7%*	*10.3%*	*10.6%*	*10.9%*	*11.1%*
Glasses — Side Shield (R) [0.35 mm PbEq]	0.37	0.38	0.38	0.38	0.38	0.38	0.38	0.38	0.38	0.38	0.38	0.38	0.38	0.38
	*6.3%*	*7.1%*	*7.1%*	*8.3%*	*7.7%*	*8.6%*	*7.7%*	*8.6%*	*7.4%*	*8.3%*	*7.1%*	*8.0%*	*7.1%*	*8.0%*
Thyroid Shield [0.5 mm PbEq]	0.46	0.46	0.46	0.47	0.47	0.47	0.47	0.47	0.46	0.46	0.46	0.46	0.45	0.45
	−*7.6%*	*‐7.4%*	*‐7.4%*	*‐6.8%*	−*7.0%*	−*6.6%*	−*7.0%*	−*6.8%*	−*7.8%*	−*7.6%*	−*8.8%*	−*8.6%*	−*9.8%*	*‐9.4%*
Apron [0.35 mm PbEq]	0.39	0.37	0.39	0.39	0.39	0.39	0.39	0.39	0.38	0.38	0.37	0.38	0.37	0.37
	*12.0%*	*6.9%*	*11.4%*	*11.4%*	*10.9%*	*11.1%*	*10.3%*	*10.6%*	*8.6%*	*9.1%*	*6.9%*	*7.7%*	*5.4%*	*6.3%*

AF, Archer’s Fit; DAK, Detector Air Kerma; LI, Linear Interpolation; PbEq, Lead Equivalency.

Per cent error between vendor/manufacturer reported lead equivalence and calculated lead equivalence using test tool is provided underneath each calculated lead equivalence.

For sake of brevity, only the unfiltered beam condition results are published in the tables. The results were similar for the 1‐mm copper filtered x‐ray beam condition.

## 4 DISCUSSION

We demonstrated that our test tool and methodology is capable of measuring the lead equivalency of radiation protection apparel by validating two known [measured] lead thicknesses. The minimum and maximum percent error values were largest when testing at 60 kVp [−9.7%, 7.1%]. Over all other beam energies, the minimum and maximum values were [−2.2%, 4.4%] for the validation results.

Most of the measured lead equivalences for the radiation protection apparel exceeded the quoted amount from the vendors/manufacturers with the exception of one of the materials. The sidepiece from the lead glasses measured across energy ranges and spectrums to be lower by 22 to 25% than the original quoted amount from the vendor. After follow‐up with the vendor, it was determined that the document provided was for newer versions of the side shields for the glasses that have a lead equivalence of 0.5 mm. Older versions of the lead glasses were made with 0.35 mm lead and confirmed to match with the manufacturer dates of the serial numbers of the glasses. With the updated information from the vendor, the sidepiece of lead measured to be greater in lead equivalence by 7 to 11% across energy levels and beam conditions.

When following this methodology, it is possible to modify the test tool’s number of squares and the thickness of each square; however, when using linear interpolation to calculate the lead equivalence, one will run into issues if there are too few foil squares or too large of a difference in thickness between squares. This can be seen when looking at the results from linear interpolation and the fitted data to Archer’s equation as the largest discrepancies between the two methods are seen between the 0.8 mm and 1.0 mm step.

With regards to picking a digital x‐ray system to use, most systems should be able to provide “FOR PROCESSING” images or *original data* that have a linear relationship between mean pixel value and detector air kerma. Ultimately, knowing the mean pixel values to detector air kerma relationship is what matters. This information can easily be obtained from the vendor’s manuals or from contacting the vendor. NEMA/MITA XR 30‐2016 — Quality Control Tools for Digital Projection Radiography^16^ has pushed for manufacturers to provide means to access and export original data in a nonproprietary format. If the relationship between original data to image receptor air kerma includes a nonlinear relationship, the vendor is supposed to provide an inverse conversion function to enable linearized data.

Particular attention also needs to be paid to the uniformity of the detector either by measurement or visual inspection to make sure there are not gross nonuniformities. With gross nonuniformities within the images, there can be significant under‐ or overestimation of the lead equivalence.

Despite only using one type of digital x‐ray system, this process is vendor independent. Some vendor’s systems are even capable of making ROI measurements on the *original data* images on the system itself which streamlines the whole process.

The geometry of this setup does not follow the geometrical design of any of the current standards mentioned above. However, it still can be used as a tool to effectively evaluate the attenuation properties of the radiation protection apparel.

## 5 CONCLUSIONS

We have validated the use of an inexpensive test tool using commercially available lead foil tape in conjunction with a digital x‐ray system for determining the lead equivalency of radiation protection apparel. It can serve as a useful tool to measure the attenuation properties in terms of lead equivalence for materials under different energy ranges and beam conditions. The methodology is equipment independent and has some prerequisites. It requires an x‐ray system that can provide adequate accuracy and reproducibility results, known lead thicknesses for the test tool, detector uniformity without gross nonuniformities, known pixel values to detector entrance dose relationship, and the ability to draw ROIs on the acquired images.

## CONFLICT OF INTEREST

The authors have no conflict of interest to declare.
